# Linguistic Validation of the LupusQoL for the Assessment of Quality of Life in Iranian Patients with Systemic Lupus Erythematosus

**DOI:** 10.1155/2014/151530

**Published:** 2014-09-09

**Authors:** Naeimehossadat Hosseini, Zahra Sayed Bonakdar, Ali Gholamrezaei, Leila Mirbagher

**Affiliations:** ^1^Medical Students' Research Center, Isfahan University of Medical Sciences, Isfahan, Iran; ^2^Department of Internal Medicine, Isfahan University of Medical Sciences, Isfahan, Iran; ^3^Poursina Hakim Research Institution, P.O. Box 81465-1798, Isfahan, Iran

## Abstract

*Objectives.* We evaluated the psychometric properties of the Persian LupusQoL for the evaluation of quality of life in Iranian systemic lupus erythematosus (SLE) patients. *Methods.* The LupusQoL was translated to Persian language. Patients with SLE (*n* = 78) completed the LupusQoL and the Short-Form Health Survey (SF-36). Disease activity and cumulative disease damage were assessed with standard indices. The psychometric properties of the scale were evaluated. *Results.* The Cronbach's alpha was 0.97 for the total LupusQoL (above 0.8 for subscales). There were strong corrected item-total (*r* > 0.4), item-subscale (*r* ≥ 0.5), and subscale-total correlations (*r* > 0.6), as well as intersubscale correlations (*r* > 0.5). Patients with active disease and patients with disease damage index of ≥1 had lower scores in domains of planning, emotional health, burden to others, and body image than patients with inactive disease and those with no disease damage, respectively (*P* < 0.05). The LupusQoL and the SF-36 correlated well regarding comparable domains (*r* > 0.4). *Conclusion.* The psychometric characteristics of the Persian version of LupusQoL questionnaire are acceptable in Iranian population. This instrument can be used to evaluate quality of life in Iranian SLE patients.

## 1. Introduction

Systemic lupus erythematosus (SLE) is a chronic disease with relapsing-remitting periods leading to high morbidity and disability in many of the patients [1]. A comprehensive evaluation of the disease state should contain disease activity, cumulative damage, and patients' quality of life (QoL) [2]. SLE specifically impairs QoL from physical, emotional, and social aspects. Therefore, according to international consensus recommendations on outcome measures in rheumatology clinical trials, QoL is among the major domains that should be assessed in clinical trials and cohorts on SLE [3].

There are generic and disease-specific measures to assess QoL. Generic measures, such as the Short-Form Health Survey-36 (SF-36), assess the patient condition in a more comprehensive manner and allow comparison between different groups (patients and healthy individuals). However, these measures are not focused on aspects of a specific disease and might not be sensitive to important clinical changes during treatment. Therefore, disease specific measures are developed not only to focus on those aspects of life being affected by the disease but also to evaluate the efficacy of different treatments [4].

In terms of the QOL in SLE patients, some disease-specific instruments are developed and applied in various studies up to now including the SLEQoL [5], SLE symptom checklist [6], L-QoL [7], LupusQoL [8], and some other instruments. The LupusQoL questionnaire is widely used according to relatively better psychometric characteristics compared with other measures [9, 10]. The questionnaire was initially developed in UK and was then validated for use in the United States and Canada. It is also translated into several other languages [11–14].

There is no specific instrument designed to assess QoL of SLE patients in Iranian population. Translation process of a questionnaire, specifically QoL questionnaires, should be as precise as the developing process and should be done on the basis of standard linguistic validation methods. The aim of this study was to linguistically validate the LupusQol and to evaluate its psychometric characteristics in Iranian population.

## 2. Materials and Methods

### 2.1. Patients and Settings

This linguistic validation study was conducted on women with SLE referring to an outpatient clinic of rheumatology in Isfahan City (Iran) between January and July 2013. After a primary report [15], we further continued the study with a relatively larger sample of patients with more precise data, including data of a generic QoL measure for comparisons. Known SLE patients, according to the American College of Rheumatology (ACR) revised criteria for SLE [16], who were at least 18 years of age and had no major cognitive deficits that would interfere questionnaire completion were consecutively included into the study. The study was approved by ethical committee of the Isfahan University of Medical Sciences and oral consent was obtained from the patients.

### 2.2. Linguistic Validation of the LupusQol

The LupusQol questionnaire is developed by McElhone and colleagues to specifically evaluate QoL of the SLE patients. It consists of 34 items in 8 different domains evaluating physical health (8 items), emotional health (6 items), body image (5 items), pain (3 items), planning (3 items), fatigue (4 items), intimate relationship (2 items), and burden to others (3 items) in the preceding 4 weeks. The answers are scored through a 5-point Likert response format from 0 to 4. To facilitate analysis and result interpretation, the final score is then transformed from 0 to 100 scale in which a higher score demonstrates a better QoL state [8]. After permission from the original developer, linguistic validation of the LupusQoL was performed by the standard method of forward-backward translation [17]. We followed the following stages: (1) conceptual definition of each item, (2) forward translation, (3) backward translation, (4) pilot study to assess cognitive debriefing and clinician's review, and (5) proofreading. The team members in validating process consisted of a rheumatologist, four professional translators who were also physicians, and the main investigators. In a pilot study with 10 participants, patients completed the translated LupusQoL and responded to a set of questions that assessed the level of difficulty and readability of the questionnaire. Based on the pilot study, the final Persian version of the LupusQoL (LupusQoL-P) was produced and used in the main study.

### 2.3. Assessments

All the patients were visited by a rheumatologist. Data including age, education level, SLE duration, comorbidities, and laboratory data were gathered by reviewing patients' documents interview. Disease activity, cumulative disease damage, and QoL were assessed by the following standard measures.

The Systemic Lupus Erythematosus Disease Activity Index 2000 (SLEDAI-2K) was used to measure the SLE activity. The SLEDAI-2K covers clinical (16 items) as well as laboratory (8 items) variables covering events in the preceding 10–30 days (the 30-day version was applied). Based on the total score, disease activity is divided into inactive (SLEDAI-2K < 6) and active (SLEDAI-2K ≥ 6) disease state [18].

The Systemic Lupus International Collaborative Clinics/American College of Rheumatology Damage Index (SDI) was used to estimate the accumulated damage since the onset of the disease. The SDI evaluates 12 organs and lupus-related complications as well as treatment-related complications. Damage is assessed only if it is persistent for at least 6 months [19].

The Short-Form Health Survey (SF-36) was applied to measure general QoL. The SF-36 contains 36 items with Likert-type as well as binary response questions. The instrument yields an 8-scale profile of physical and emotional health including physical functioning, role-physical, bodily pain, general health, vitality, social functioning, role-emotional, and mental health. All scores are converted to a range of 0 to 100 in which higher scores indicate better QoL status [20].

### 2.4. Statistical Analyses

Data were analyzed using the SPSS software (version 16.0, SPSS Inc., Chicago, IL). Reliability was assessed by determining the internal consistency (Cronbach's alpha) of the total LupusQoL and the subscales. Convergent validity was assessed by determining corrected item-subscale and subscale-total correlations as well as intersubscale correlations. Concurrent validity was assessed by testing the correlations between the LupusQoL and the SF-36 comparable domains. Discriminant validity was assessed by comparison between patients with active and inactive disease, as well as comparison between patients with no disease damage and patients with damage index of ≥1. In all analyses, a *P* value (two-tailed) of less than 0.05 was considered statistically significant.

## 3. Results

### 3.1. Patient Characteristics

One hundred SLE patients were invited to participate in this survey, of whom six patients refused to enroll in the study and six other patients partially filled the questionnaires and therefore were excluded. Ten patients were enrolled in the pilot study and 78 patients were enrolled in the main survey. The data of the 10 patients from the pilot study was not considered for the psychometric analyses.  Table 1 summarizes the demographic and clinical characteristics of the patients.

### 3.2. The LupusQoL Psychometric Characteristics

Participants had responded to 99% of the LupusQoL items. Missing data varied from 0 to 2 (2.5%). Ceiling effect was observed for item 3 (69.2%) and item 33 (59%). No floor effect was observed.

#### 3.2.1. Internal Consistency

The internal consistency and item analysis are summarized in Table 2. The Cronbach's alpha was 0.97 for the total LupusQoL and from 0.82 to 0.93 for the subscales. Total alpha remained stable at 0.97 by deleting each of the items. Deleting none of the items increased the corresponding subscale alpha. Corrected item-total correlations were above 0.4 for all items except items 10 and 33 (*r* = 0.39).

#### 3.2.2. Convergent Validity

The corrected item-subscale correlations were ≥0.5 for all items (range from 0.50 to 0.91). All subscales had the corrected subscale-total correlations of >0.6 (range from 0.61 to 0.89, Table 2). Also, there were strong correlations between different subscales (range from 0.51 between pain and intimate relationship to 0.81 between pain and fatigue).

#### 3.2.3. Concurrent Validity

Correlations between the LupusQoL and the corresponding SF-36 domains are presented in Table 3. For the 4 comparable domains, the 2 measures correlated well; *r* = 0.80 for physical health/physical functioning, *r* = 0.70 for emotional health/mental health, *r* = 0.73 for pain/bodily pain, and *r* = 0.55 for fatigue/vitality. Other expected correlations were also present at *r* > 0.4.

#### 3.2.4. Discriminant Validity

Comparison of patients' QoL according to disease activity and cumulative disease damage is summarized in Table 4. Patients with active disease had significantly lower scores in domains of planning (*P* = 0.041), emotional health (*P* = 0.044), and body image (*P* = 0.019) than patients with inactive disease. Also, patients with damage index of ≥1 had significantly lower scores for planning (*P* = 0.002), burden to others (*P* = 0.039), and body image (*P* = 0.012) than those with no disease damage.

## 4. Discussion

Quality of life is of the most important measurable outcomes in patients with SLE. We aimed to linguistically validate the LupusQoL and determine its psychometric characteristics in Iranian SLE patients with Persian language. Our results revealed that the LupusQoL-P is a valid and reliable disease-specific instrument for assessing QoL in Iranian SLE patients. In this survey, patients' cooperation was acceptable and there was limited number of missing data for most of the items implying good acceptance and understanding of patients towards the translation. In our study, no floor effect was observed. The minimal observed ceiling effect could be explained by the homogenicity of the studied sample. The calculated Cronbach's alpha for the whole questionnaire (0.97) and its different domains (0.82 to 0.93) demonstrates a good internal consistency. Lack of change in alpha coefficients following removal of each item showed that all the items were consistent with the whole questionnaire. In the main questionnaire designed by McElhone et al., alpha coefficient ranged from 0.88 to 0.96 for different domains of the instrument [8]. Also the American version of the LupusQoL reported alpha coefficient between 0.85 and 0.94 [21]. Surveys on other versions of the questionnaire such as French, Chinese, and Italian translations also reported similar results [11–14].

Consistently with other validation surveys [11–14], we found that each item of the LupusQoL-P has a high correlation with its corresponding domain. Also, there was strong correlation between the LupusQoL-P and the corresponding SF-36 domains, especially those of physical health, emotional health, pain, and fatigue. These results indicate appropriate convergent and concurrent validity of the scale. The validity of the LupusQoL against generic QoL measures is also confirmed by other validation studies [11–14].

We expected to find a difference between patients with and without active disease state and also between those with and without disease damage regarding their QoL. In our study, several but not all domains of the LupusQoL-P were affected by disease activity and disease damage including planning, burden to others, emotional health, and body image. Although the original study has reported weak correlations between the scores of the LupusQoL domains and disease activity and damage scores, later linguistic validation studies have shown appropriate discriminant validity of the scale. In this regard, Conti and colleagues found a difference in the domains of physical health, planning, burden to others, and fatigue between patients with and without active disease [11]. Wang and colleagues found a difference in almost all domains of the LupusQoL-China (except body image and burden to others) between patients with different disease activity and damage state [12]. Also, Devilliers et al. reported a difference in domains of physical health, pain, and intimate relationship between patients with active and inactive disease [14]. Differences among the previous studies might be related to differences in patients' characteristics and the disease activity measures. To evaluate the discriminant validity of the scale, a heterogeneous sample of patients with various stages of the disease activity and damage is required. It should be noted that our study sample size was small in this regard and not many of our patients had active disease or considerable disease damage. Therefore, further studies are warranted in this regard.

## 5. Conclusions

The Persian version of the LupusQoL (LupusQoL-P) has appropriate validity and reliability among Iranian patients with SLE. While this linguistic validation study is done in a single care center, further multicenter studies are needed to evaluate Iranian SLE patients' QoL and contributing factors. Such data would be useful for healthcare providers in designing and implementing strategies to improve QoL of the patients.

## Figures and Tables

**Table 1:** Characteristics of the study participants (*n* = 78).

	Mean ± SD or number (%)
Age, year	36.8 ± 10.1
Education, year	11.2 ± 3.8
Disease duration, year	8.2 ± 3.8
SLEDAI-2K	3.2 ± 3.6 (median 2)
SLEDAI-2K ≥ 6	16 (20.5)
SDI	0.47 ± 0.86 (median 0)
SDI ≥ 1	25 (32)
Comorbidities	
Hypertension	19 (24.3)
Diabetes	2 (2.6)
Kidney disease	27 (34.6)
Other rheumatologic diseases	11 (14.1)

SLEDAI-2K: Systemic Lupus Erythematosus Disease Activity Index 2000; SDI: Systemic Lupus International Collaborative Clinics/American College of Rheumatology Damage Index.

**Table 2:** Cronbach's alpha for the total scale and its subscales' and item analysis.

	Subscale Alpha and if item deleted	Total Alpha if item deleted	Corrected item-subscale correlation	Corrected item-total and subscale-total correlations
**Physical health**	**0.93**			**0.83**
Items 1–8	0.91–0.93	0.97	0.60–0.87	0.62–0.87
**Pain**	**0.85**			**0.81**
Items 9–11	0.72–0.85	0.97	0.67–0.80	0.39–0.88
**Planning **	**0.89**			**0.84**
Items 12–14	0.82–0.87	0.97	0.77–0.82	0.78–0.89
**Intimate relationship**	**0.92**			**0.64**
Items 15 and 16	—	0.97	0.86	0.78 and 0.64
**Burden to others**	**0.93**			**0.74**
Items 17–19	0.86–0.90	0.97	0.80–0.91	0.79–0.88
**Emotional health**	**0.93**			**0.75**
Items 20–25	0.91–0.93	0.97	0.72–0.89	0.61–0.84
**Body image**	**0.84**			**0.73**
Item 26–30	0.80–0.83	0.97	0.60–0.69	0.48–0.83
**Fatigue**	**0.82**			**0.86**
Item 31–34	0.74–0.82	0.97	0.50–0.73	0.39–0.89

**Table 3:** Concurrent validity of the LupusQoL-P in comparison with the Short-Form Health Survey 36 (SF-36).

LupusQoL domain	SF-36 domain	Pearson correlations
Physical health	Physical functioning	0.80
	Role physical	0.72

Pain	Bodily pain	0.73

Planning	Physical functioning	0.72
	Social functioning	0.71

Intimate relationship	Social functioning	0.56
	Mental health	0.56

Burden to others	Physical functioning	0.63
	Social functioning	0.54

Emotional health	Mental health	0.70
	Role emotional	0.56

Body image	Social functioning	0.52
	Role emotional	0.48

Fatigue	Vitality	0.55

**Table 4:** Discriminant validity of the LupusQoL according to disease activity and damage.

	SLEDAI-2K < 6 *n* = 62	SLEDAI-2K ≥ 6 *n* = 16	*P*	SDI = 0 *n* = 53	SDI ≥ 1 *n* = 25	*P*
Physical health	70.7 ± 26.3	70.3 ± 22.6	0.946∗	73.5 ± 24.0	64.6 ± 27.9	0.262∗
Pain	70.0 ± 27.4	67.2 ± 32.1	0.815∗	72.9 ± 26.6	62.3 ± 30.7	0.131∗
Planning	75.5 ± 28.6	63.3 ± 26.3	0.041∗	79.8 ± 25.8	59.3 ± 29.0	0.002∗
Intimate relationship	74.5 ± 29.7	63.8 ± 26.1	0.129∗	76.3 ± 27.8	65.6 ± 32.1	0.148∗
Burden to others	55.5 ± 33.2	46.6 ± 35.0	0.348∗	60.0 ± 30.0	40.6 ± 37.1	0.039∗
Emotional health	55.0 ± 26.6	41.0 ± 22.0	0.044∗	55.3 ± 26.5	45.6 ± 24.8	0.137∗
Body image	67.3 ± 27.1	51.8 ± 23.4	0.019∗	68.8 ± 27.0	52.9 ± 23.9	0.012∗
Fatigue	65.7 ± 24.9	57.7 ± 22.0	0.178∗	66.7 ± 24.3	58.5 ± 24.3	0.161∗

SLEDAI-2K: Systemic Lupus Erythematosus Disease Activity Index 2000; SLICC/ACR: Systemic Lupus International Collaborative Clinics/American College of Rheumatology Damage Index.

∗Mann-Whitney *U* test.
